# KF-finder: identification of key factors from host-microbial networks in cervical cancer

**DOI:** 10.1186/s12918-018-0566-x

**Published:** 2018-04-24

**Authors:** Jialu Hu, Yiqun Gao, Yan Zheng, Xuequn Shang

**Affiliations:** 1 School of Computer Science, Northwestern Polytechnical University, West Youyi Road 127, Xi’an, 710072 China; 2 Centre of Multidisciplinary Convergence Computing, School of Computer Science, Northwestern Polytechnical University, Dong Xiang Road 1, Xi’an, 710129 China

**Keywords:** 16s rRNA, Host-microbial network, Cervical carcinoma

## Abstract

**Background:**

The human body is colonized by a vast number of microbes. Microbiota can benefit many normal life processes, but can also cause many diseases by interfering the regular metabolism and immune system. Recent studies have demonstrated that the microbial community is closely associated with various types of cell carcinoma. The search for key factors, which also refer to cancer causing agents, can provide an important clue in understanding the regulatory mechanism of microbiota in uterine cervix cancer.

**Results:**

In this paper, we investigated microbiota composition and gene expression data for 58 squamous and adenosquamous cell carcinoma. A host-microbial covariance network was constructed based on the 16s rRNA and gene expression data of the samples, which consists of 259 abundant microbes and 738 differentially expressed genes (DEGs). To search for risk factors from host-microbial networks, the method of bi-partite betweenness centrality (BpBC) was used to measure the risk of a given node to a certain biological process in hosts. A web-based tool KF-finder was developed, which can efficiently query and visualize the knowledge of microbiota and differentially expressed genes (DEGs) in the network.

**Conclusions:**

Our results suggest that *prevotellaceade*, *tissierellaceae* and *fusobacteriaceae* are the most abundant microbes in cervical carcinoma, and the microbial community in cervical cancer is less diverse than that of any other boy sites in health. A set of key risk factors *anaerococcus*, *hydrogenophilaceae*, *eubacterium*, *PSMB10*, *KCNIP1* and *KRT13* have been identified, which are thought to be involved in the regulation of viral response, cell cycle and epithelial cell differentiation in cervical cancer. It can be concluded that permanent changes of microbiota composition could be a major force for chromosomal instability, which subsequently enables the effect of key risk factors in cancer. All our results described in this paper can be freely accessed from our website at http://www.nwpu-bioinformatics.com/KF-finder/.

## Background

Cervical cancer is the second most common cancer in women [1]. Over 500,000 women worldwide die of cervical cancer each year [2]. It is known that a persistent human papillomavirus (HPV) infection appears to be one of major causes of cervical carcinoma. HPV-16 or HPV-18 has been found in more than 70% of cases [3–5]. These oncogenic HPVs are also common risk factors in some other cancers, such as head and neck cancers [6]. However, there are still gaps in the knowledge of cervical cancer to answer the question of why HPV is necessary to cause cell carcinoma, although it is not a sufficient requirement [1, 7].

Thanks to the advent of high-throughput technologies, researchers are able to analyze the cervical carcinogenesis at the genomic level using sequencing data [8]. Genome-wide association studies and subsequent meta-analyses showed that differentially expressed genes (DEGs) in cervical cancer are more likely to locate in the region of frequent chromosomal aberration [9–12]. It indicates that cancer may strongly associate with the chromosomal instability [13]. A recent study suggests that microbiota might play important roles in the development of cervical cancer [14]. There exists a significant difference in microbiota’s diversity between non-cervical lesion (NCL) HPV negative women and these with cervical cancer. Further, compared to the microbial community in NCL-HPV negative ones, these in cervical cancer samples have higher variation within groups. All these findings implicate that cervical microbiota is an important clue in the research of the cervical cancer pathology. In order to understand how the microbial community interplay with host genes and cause cell carcinoma in the molecular level, more and more research groups make efforts of identify key factors, also known as cancer-causing agents, which can drive the progress of cervical carcinogenesis.

Microbiota is a possible suspect causing the frequent gains and losses in chromosome. It is abundantly distributed in women cervices. They are involved in many of the host’s normal life processes, but also can destroy the host’s normal gene regulatory network by gene transfer, which may activate oncogene expression and lead to cancer [15]. Therefore, many researchers take efforts to study how the human microbiota cause structural variation of human genomes and alter the immune system and metabolic system to support the development of cervical pathogenesis [16]. Permanent changes of microbiota may be a major cause of chromosomal instability, subsequently discharge the tumor suppressor gene retinoblastoma (RB) and tumor protein TP53. Some association measures can be used to build a covariance network for microbes and host genes [17]. Host-microbial networks provide a systematic way to study the regulation system between microbiota and host genes [18]. However, the role of host response to the change of microbiome in cervical cancer is still unknown. And there are only a few public tools specifically designed for analyzing host-microbial networks [19–21]. Therefore, there is a pressing demand to develop fast and efficient computational tools to examine how microbiota regulate the gene expression, chromosomal instability and cell carcinoma.

As a remedy for these limitations, we proposed a new computational framework to identify the key risk factors using 16s rRNA and gene expression data of 58 squamous and adenosquamous cell carcinoma in uterine cervix. A series of meta-analyses was performed, which include error correction, spearman rank correlation, differential expression analysis, and bi-partite betweenness centrality. A web-based tool KF-finder was developed, which can provide users a fast-and-easy way to query and visualize the knowledge of microbiota and genes in cervical cancer. Further, a set of novel risk factors were identified that may give helpful suggestions for these researchers focusing on drug design and pharmacology.

## Methods

In order to investigate gene expression and microbiome composition in cervical cancer, we collected 133 squamous and adenosquamous cell carcinoma samples, 58 out of which were used for microbial DNA library preparation. The 16s rRNA sequencing was performed using Illumina MiSeq. Human gene expression was quantified using WG-6 BeadArray.

### OTU assignment

Each 16s sequence was assigned to an operational taxonomic unit (OTU). To count the reads number for each OTU (microbe), 16s sequences obtained from MiSeq were aligned to the reference Greengene OTU builds. The Qiime script assigne_taxonomy.py (see more at http://qiime.org/scripts/assign_taxonomy.html) was performed in the data processing. Reference sequences are pre-assigned with OTU described in the id_to_taxonomy file. Any sequence alignment tools, such as uclust, SortMeRNA, blast, RDP, Mothur etc, can be called by the assign_taxonomy script for the sequence alignment between the 16s sequences and reference sequences. For example, the script will assign taxonomy with the uclust consensus taxonomy assigner by default using the following command, *assign_taxonomy.py -i repr_set_seqs.fasta -r ref_seq_set.fna -t id_to_taxonomy.txt*. OTU redundancy matrix was normalized from the sequence number of each sample. Since these less abundant microbes are unlikely to be a destroying force for host immune system, we selected the top-259 most abundant OTUs for further studying.

### Comparison with the controls

To study the remarkable difference of microbiota between cancer cases and the controls, we compared our 16s raw data to those data from 300 healthy human subjects released by Human Microbiome Project (HMP) [22] (http://www.hmpdacc.org). To find a map between OTUs from our data and OTUs from healthy data, a commonly used alignment tool *blastn* was performed to compare their representative sequences. These pairs with evalue <1e-5 and pident >80% were used for establishing the map. These OTUs matched with a same OTU in HMP were collapsed into one OTU. The Qiime scripts were performed to analyze the 16s raw data [23].

### Calculation of correlation

Abundant microbes and DEGs were selected for reconstructing host-microbial networks. DEGs in cervical cancer were collected from published data [9], which were verified in five cohorts of tumor and normal samples. Hence, the DEGs are more reliable than these obtained from only one cohort. The spearman rank correlation method was employed to calculate the correlation between each pair of nodes. Note that, the gene expression data and 16s rRNA were tested on the same sample. Therefore, the spearman correlation in the network makes sense. In contrast to pearson correlation, spearman correlation coefficient can efficiently avoid the environmental noise and experimental errors caused from the non-uniform samples.

### Error correction

**Fig. 1:**
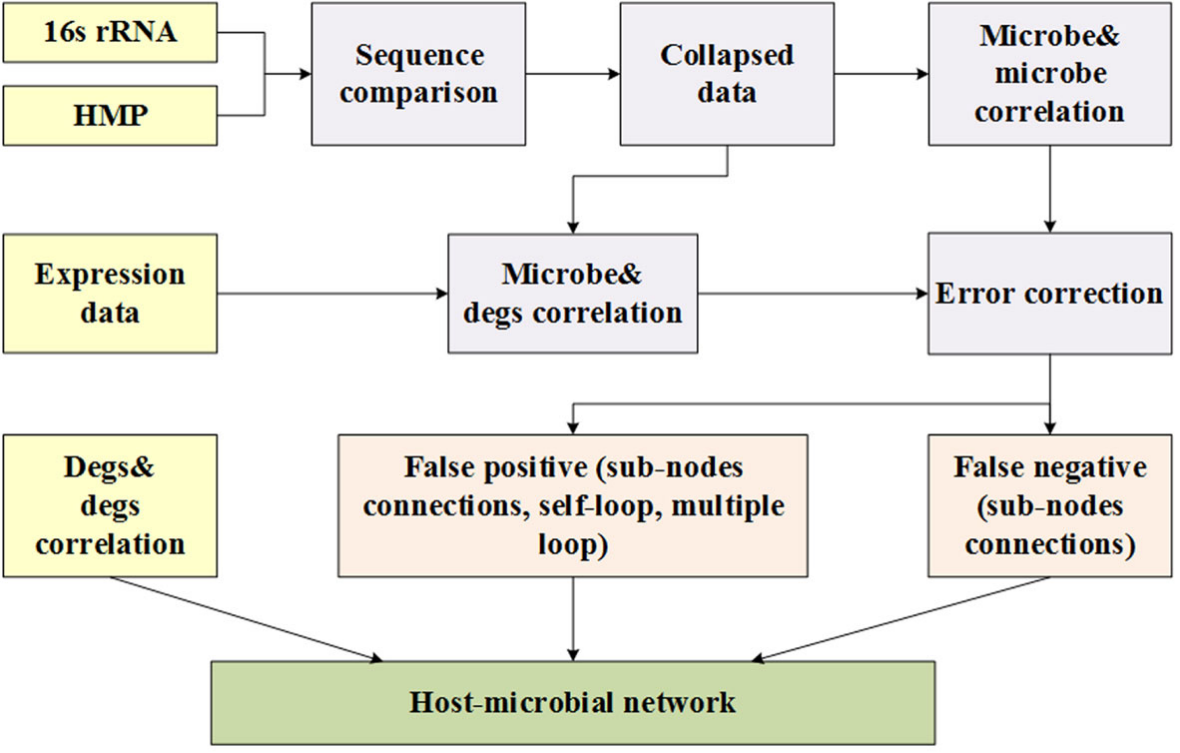
A workflow of the reconstruction of host-microbial network. Through the comparison between 16s rRNA and HMP data, each sequence was mapping to an operational taxonomic unit (OTU). Error correction was performed for these false positive and false negative nodes, which were detected according to the coherence of regulation and correlation

To improve the confidence of the host-microbial network calculated by spearman correlation, we removed these edges that are less likely to be a true one (false positive errors) and added some new edges that are very likely to correlate with each other (false negative errors). The false positive edges include two scenes: 1) these negatively correlated edges that connected two interactors with a same type of regulation (i.e. both of them are up regulated or down regulated); 2) these positive correlated edges that connected two interactors with different types of regulation (i.e. one is up regulated, the other down regulated); 3) self-loops; 4) multiple loops. All these false positive edges are removed in our network. These false negative edges are these pairs of nodes between OTUs and DEGs which satisfying two conditions: 1) the OTU was collapsed from a set of sub-nodes; 2) all these sub-nodes strongly correlated with the DEG. All these false negative edges were added in the host-microbial network. False positive and false negative edges were detected and corrected according to the coherence of regulation and correlation relationships. A workflow of the reconstruction of host-microbial network was illustrated in Fig. 1.

### Bi-partite betweenness centrality

To search for risk factors from host-microbial network, bi-partite betweenness centrality (BpBC) [24], adapted from betweenness centrality, was used to quantify the risk of a given node, written as g(v). The definition can be formatted as $g(v)=\sum _{s,t}\delta _{st}(v)/\delta _{st}$. Here, s and t are two nodes from two separate sub-networks. And *δ*_*st*_ represents the number of shortest paths from s to t, *δ*_*st*_(*v*) the number of shortest paths going through node v from s to t. Given a node v, g(v) reflects the probability of how likely a shortest path could go through v from one sub-network to another.

## Results and discussion

### Composition of the microbiota

**Fig. 2:**
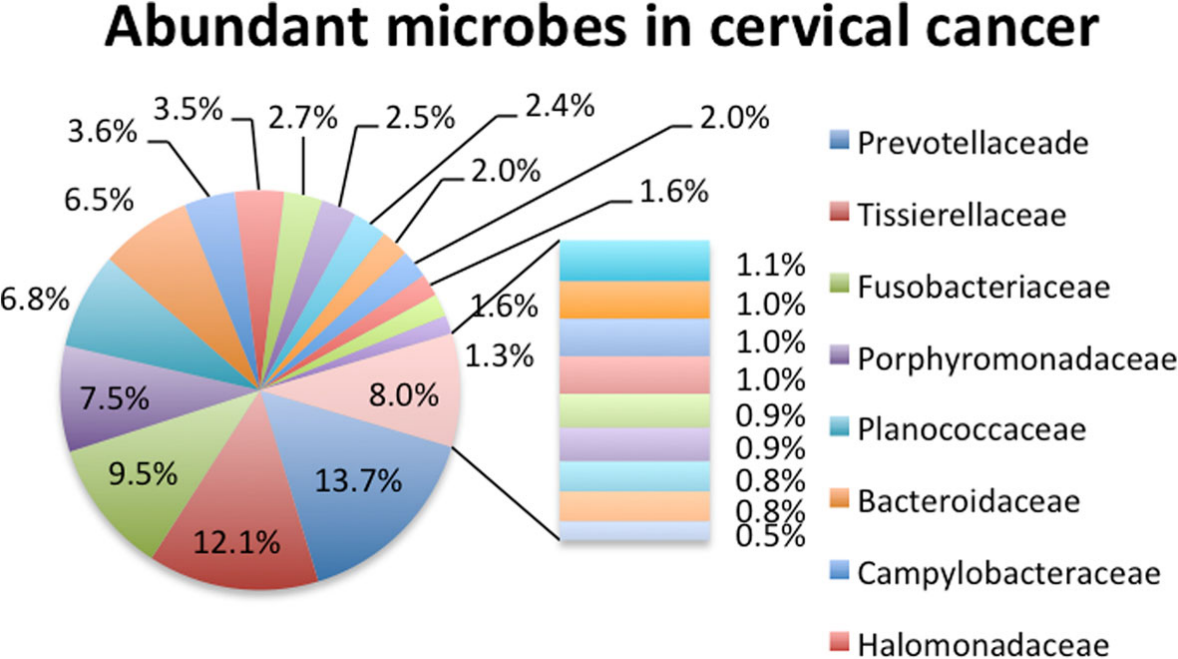
The microbial community in cervical carcinoma. Each 16s rRNA sequence was assigned to an operational taxonomic unit (OTU), and all these sequences were grouped into different categories based on their family-level OTU labels

**Fig. 3:**
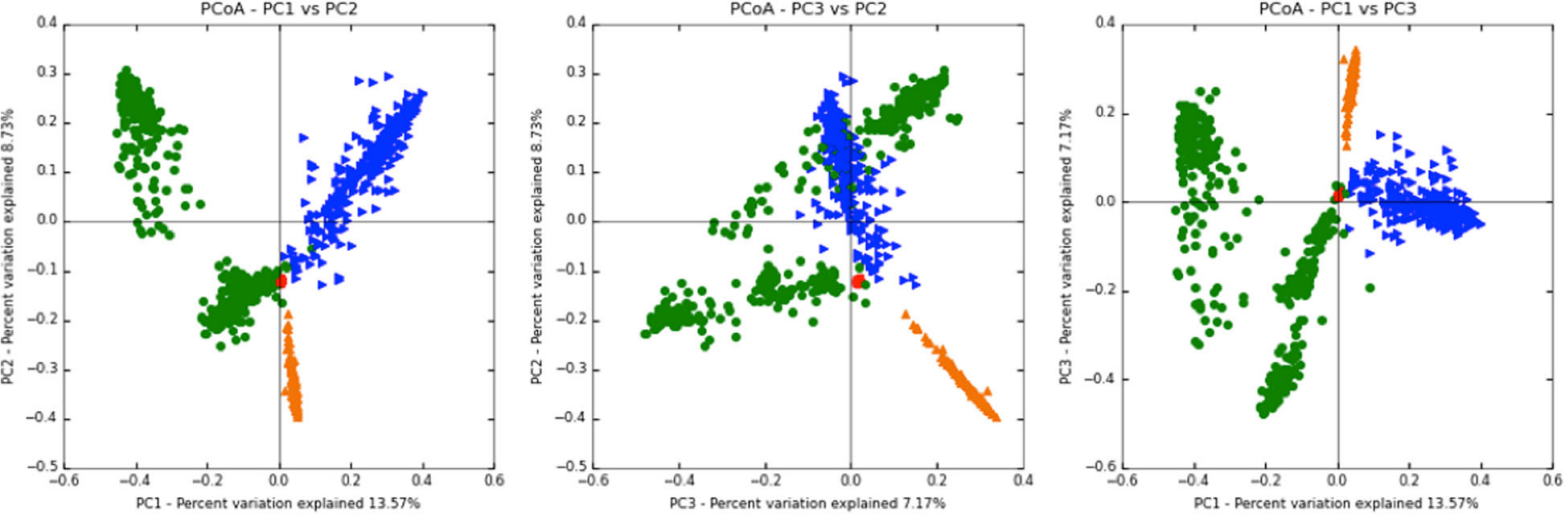
Principal Coordinates Analysis (PCoA) plot of microbial community for samples from cervical carcinoma, skin, mouth and vagina. The red, green, orange and blue dots represent samples from cervical carcinoma, skin, mouth and vagina, respectively

To study the microbial community in cervical cancer, we examined the 16s raw data of cancer cases and assigned taxonomy to each sequence. The definition of operational taxonomic unit (OTU) was used to classify groups of closely related microbiome based on sequence similarity. Reference data sets and id-to-OTU maps for 16s rRNA sequence was downloaded from the Greengenes reference OTU builds [25]. All these sequences were grouped into different categories based on their family-level OTU labels. As shown in Fig. 2, *prevotellaceade* followed by *tissierellaceae* appears to be the most abundant microbes, accounting for 13.7% of the microbiota community. There are four other groups accounting for more than 5% of the microbiota, which are *fusobacteriaceae*, *porphyromonadaceae*, *planococcaceae* and *bacteroidaceae*. Totally, twenty-six family-level OTU groups make up more than 87% of the whole community. To examine the diversity of cervical microbiota, the PCoA analysis was performed to analyze the microbial community in cervical carcinoma, skin, mouth and vagina. As shown in Fig. 3, microbiota in cervical carcinoma (red dots) is less diverse than microbiota in any other body sites. Hence, we indeed found remarkable changes of microbial composition in the cancer cases.

### Reconstruction of host-microbial network

**Fig. 4:**
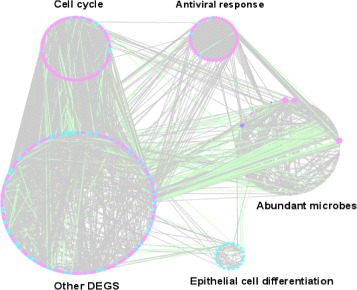
An illustration of the host-microbial network. Nodes refer to differentially expressed genes (DEGs) or abundant microbes, edges the regulation relationship between DEGs and microbes. Nodes in pink are up regulated, and these in cyan are down regulated. Edges in grey are positively correlated, and these in green are negatively correlated

A host-microbial network was reconstructed from the 16s raw data and gene expression data. Nodes in the network refer to microbes or DEGs, edges the regulation relationships between each pair of microbes. Two nodes were connected if and only if they are strongly correlated (i.e. |*γ*|>0.4 and *p*-value < 0.05). As show in Fig. 4, a network with 997 nodes was connected by 4262 edges. Nodes in the network consist of 259 microbes and 738 DEGs. We grouped all the DEGs into four categories, named as cell cycle, antiviral response, epithelial cell differential and the other DEGs, according to their function in the development of cervical cancer. The three functional DEGs groups (excluding the other DEGS) are three major densely connected sub-networks in the host-microbial networks. They are functionally enriched by GO terms cell cycle, response to virus, epithelial cell differentiation respectively. They don’t have any overlap between each pair of groups. In the whole network, 403 edges are negatively correlated, 3859 positively correlated. Negative correlation indicates inhibition between two biological subjects. In a negative correlation, one variable increases as the other decreases. Positive correlation indicates activation or co-existence between two subjects of interest. In a positive correlation, one variable increases as the other increase, or one variable decreases while the other decreases. This network integrates all the regulation relationships between host genes and microbiota.

### Risk factors in cervical cancer

The risk factors in cancer may activate oncogene expression and cause a series of functional disorder in metabolic and immune systems. In the development of cancer, the most remarkable differences between tumor and normal samples are: 1) the up-regulation of viral responses; 2) the speed-up in the progression of cell cycle; 3) the inhibition of epithelial cell differentiation. To study how microbiota regulates the viral response, cell cycle and epithelial cell differentiation, we searched for key risk factors using BpBC. These key factors are thought to be cancer-causing agents that can drive the progress of cervical carcinogenesis. Nodes that organizing communication between two cancer-related groups are more likely to be key factors. Since BpBC is such a measure to evaluate the importance of a node in the network topology, we choose these nodes in the top list of BpBC as candidates of key factors. These key factors with high BpBC value may play crucial roles in the communication between two different sub-networks.

**Fig. 5:**
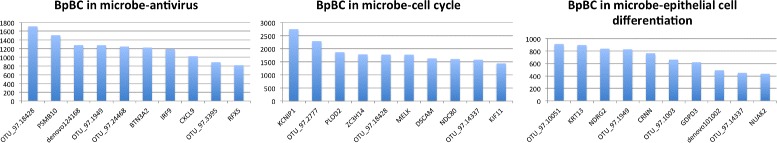
Risk factors in host-microbial network in cervical cancer. The BpBC value of each node was calculated for three pairs of different sub-networks, including microbe-antivirus, microbe-cell cycle and microbe-epithelial cell differentiation

The results show that *Anaerococcus* (labeled as OTU_97.18428) and proteasome subunit beta 10 (PSMB10) are significantly higher than the others (see in Fig. 5 left) between the sub-networks of microbe and antiviral response genes. PSMB10 was an up-regulated gene in cervical cancer. Between the sub-networks of microbe and cell cycle, KCNIP1 and Hydrogenophilaceae (labeled as OTU_97.2777) are the most important regulators (see in Fig. 5 middle). Eubacterium (labeled as OTU_97.10051) and KRT13 are the most important regulators between the sub-networks of microbes and epithelial cell differentiation (see in Fig. 5 right). It proves that the interplay between microbiota and differentially expressed genes might be the driving force that regulates the progress of cell cycle, epithelial cell differentiation and viral response.

### Query and visualization

**Fig. 6:**
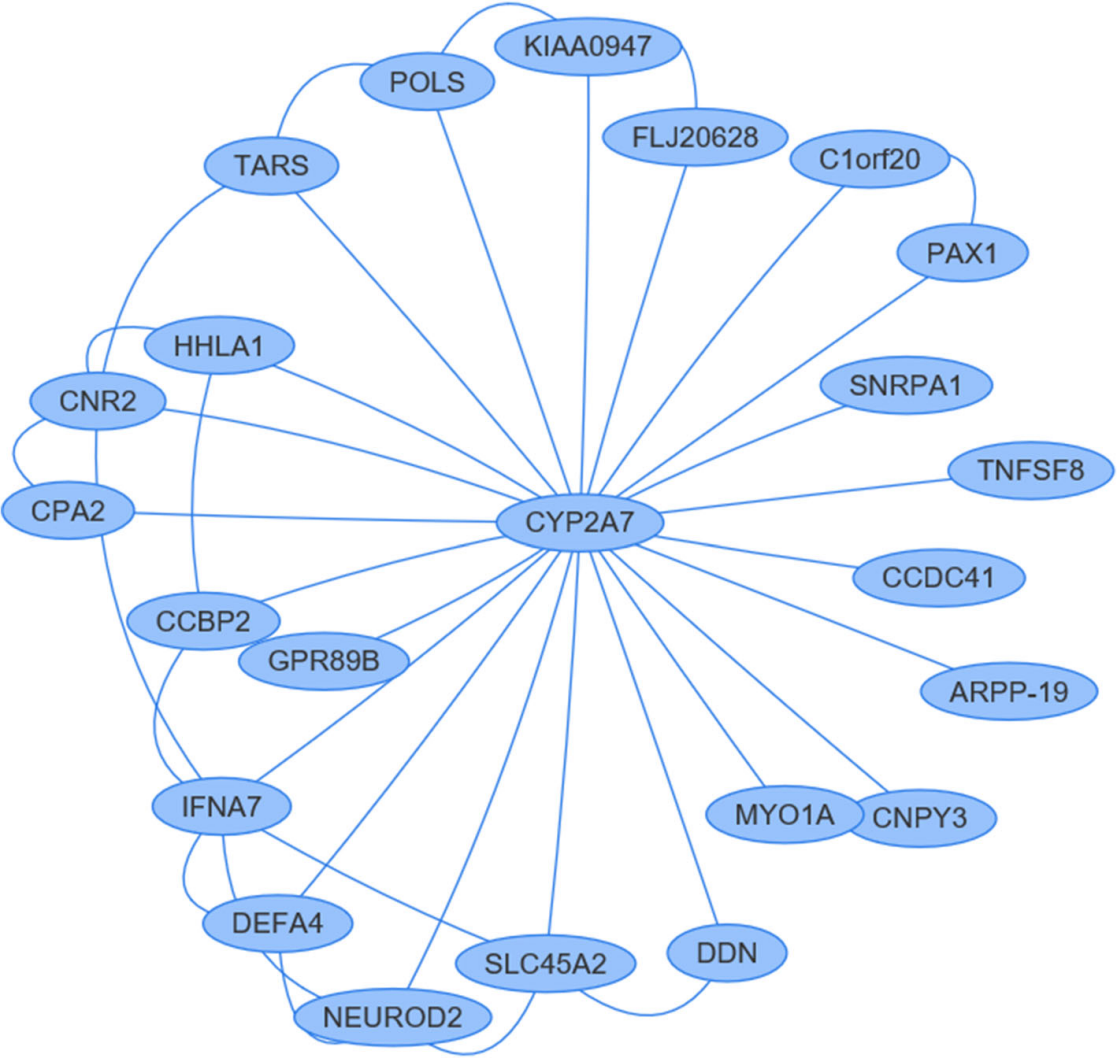
A graphic view of the induced sub-network of CYP2A7. The subnetwork includes interactions between CYP2A7 and its neighbors, interactions between its neighbors

In order to fast and easily query and visualize the host-microbial networks, we developed a web-based tool KF-finder. Multiple web programming languages were used in the development, which includes PHP, mysql and javascripts. Each node and its neighborhood in the network can be searched by a query term in the panel of Search. And the induced sub-network will be visualized in the panel of View. For example, one can input a gene symbol CYP2A7 as a query term in the Search panel. A list of nodes associated with CYP2A7 will show out in a user-friendly panel, as well as a graphic view of the induced sub-network (see in Fig. 6). Except for visualization and query, KF-finder can also sort microbes and DEGs in a decreasing order by the value of BpBC in microbe-antivirus, microbe-cell cycle or microbe-epithelial cell differentiation. Download and advanced search have been enabled on the web server. All our test datasets and results of users’ personal jobs can be downloaded. Advanced search allows us search for genes and microbes based on string patterns or value constriction. KF-finder enables us to query and visualize the knowledge of host-microbial network in a fast-and-easy way. It can be accessed at http://www.nwpu-bioinformatics.com/KF-finder/.

### A case study of PSMB10 in cervical cancer

Most vertebrates express immunoproteasomes (IPs) that possess three IFN- *γ*-inducible homologues: PSMB8, PSMB9 and PSMB10. Many studies show that expression of IP genes including PSMB10 is up-regulated in most cancer types [26]. IP genes can be expressed by non-immune cell, and that differential cleavage of transcription factors by IPs has pleiotropic effects on cell function. Indeed, IPs modulate the abundance of transcription factors that regulate signaling pathways with prominent roles in cell differentiation, inflammation and neoplastic transformation (e.g., NF-kB, IFNs, STATs and Wnt) [27]. Therefore, PSMB10 is indeed a risk factor involved in the antiviral response of cervical caner.

### A case study of KRT13 in cervical cancer

KRT13’s full name is keratin 13 in human, also known as K13 and CK13, located in a region of chromosome 17q21.2. It is a down-regulated gene in cervical carcinoma, and a risk factor that involves in the progress of uncontrolled epithelial cell differentiat,ion. Previous work suggests that the loss of K13 or low K13 mRNA expression is associated with invasive oral squamous cell carcinoma (OSCC) [28, 29]. Epigenetic alteration of K13 is one major reason resulting the inhibition of K13 in OSCC. Besides, K13 was also reported that it played a directive role in prostate cancer bone, brain and soft tissue metastases [30]. More than 1000 single nucleotide polymorphisms of K13 were found in the dbSNP database. Totally, 51 variations mentioned K13 in ClinVar, seven out of which are pathogenic. All these evidences suggest KRT13 is very likely to be a key risk factor involved in cervical cancer.

## Conclusions

In this paper, we examined the microbiota composition and gene expression in 58 squamous and adenosquamous cell carcinoma. A host-microbial network was reconstructed from the 16s rRNA and gene expression data. The main contributions of this paper can be concluded in three aspects: (1) microbial community distributed in cervical carcinoma cells is less diverse than that of other body sites; (2) a web-based tool MiteFinder was developed which enables users to query and visualize host-microbial networks, microbes and differentially expressed genes in a fast-and-easy way; (3) a set of key risk factors have been identified, which have proven to have association with cancers in several previous publications. Our results show that six groups of OTU abundantly distributed in cervical cancer samples, including *prevotellaceade, tissierellaceae, fusobacteriaceae, porphyromonadaceae, planococcaceae and bacteroidaceae*. Besides these six groups of OTU, we found that three differentially expressed genes and three microbes may be key risk factors and play crucial roles in the pathology of cervical carcinoma. All of these results suggest that permanent changes of microbiota composition might be the key driving force in the pathology of cervical carcinoma, which result in the abnormality of epithelial cell differentiation, cell cycle and viral response.
